# Association between Carotid Wall Shear Rate and Arterial Stiffness in Patients with Hypertension and Atherosclerosis of Peripheral Arteries

**DOI:** 10.1155/2018/6486234

**Published:** 2018-08-01

**Authors:** Vadim V. Genkel, Alexey O. Salashenko, Tatyana N. Shamaeva, Veronika A. Sumerkina, Igor I. Shaposhnik

**Affiliations:** Federal State Budgetary Educational Institution of Higher Education “South-Ural State Medical University” of the Ministry of Healthcare of the Russian Federation, Vorovskogo st. 64, 454092 Chelyabinsk, Russia

## Abstract

**Aim:**

To evaluate carotid wall shear rate (WSR) in association with local and regional vascular stiffness in patients with hypertension (HTN) and atherosclerosis of peripheral arteries and to study the pattern of change of WSR in patients with HTN with increasing severity of peripheral artery atherosclerosis.

**Materials and Methods:**

Study involved 133 patients with HTN, 65 men and 48 women, aged in average 57.9±10.8 years. All patients were divided into four groups in accordance with ultrasound morphologic classification of vessel wall. Duplex scanning of carotid and lower limb arteries was performed. Carotid-femoral (cfPWV) and carotid-radial (crPWV) pulse wave velocity (PWV) were measured. Local carotid stiffness was evaluated by carotid ultrasound.

**Results:**

WSR of patients with plaques without and with hemodynamic disturbance was 416±128 s^−1^ and 405±117 s^−1^, respectively, which was significantly less than the WSR in patients with intact peripheral arteries – 546±112 s^−1^. Decreased carotid WSR was associated with increased crPVW, cfPWV, Peterson's elastic modulus, decreased distensibility, and distensibility coefficient.

**Conclusion:**

In patients with HTN and atherosclerotic lesions of peripheral arteries, it is registered that the carotid WSR decreased with increasing severity of atherosclerosis. Decreased carotid WSR is associated with increased local carotid stiffness, regional vascular stiffness of muscular, and elastic vessels.

## 1. Introduction

Atherosclerosis is a systemic chronic inflammatory disease predominantly affecting muscular and elastic arteries, characterized by autoimmune response to arterial wall damaging with development of subintimal accumulation of lipids, immunocompetent cells, and smooth muscle cells [1]. The key event in initiation and development of atherosclerosis is endothelial injury [2]. State of endothelium is integrative indicator of all atherogenic and atheroprotective factors to which this individual organism is exposed [3]. The vessel wall continuously undergoes impact of biomechanical forces; the application point of it is both intima and media. One of the most important forces is wall shear stress (WSS) that is tangential oriented and emerged under the action of viscosity generated by moving stream of blood to endothelial cells [4, 5]. Currently, the great role of WSS in regulation of endothelial cells state is established. There is important role of the WSS in the regulation of the endothelial cell state mediated by the mechanotransduction of the signal into the cell that determines change of endothelial cell phenotype [6–8]. It has been established that decreased WSS is a proatherogenic factor leading to activation of endothelium with increasing of subintimal accumulation of lipids and development of atheroma [9, 10]. In addition, it is reliably established that various risk factors damage not only intima but also media. Today, there is evidence that forces acting not only from the lumen but from inside, i.e., from media, have an extremely important influence to endothelium. Increased stiffness of subendothelial matrix induces increasing of permeability of endothelial layer and increasing of transendothelial migration of immunocompetent cells to subintimal space [11, 12].

Thereby, atherosclerotic plaque formation is a result of interactions between systemic atherogenic risk factors and features of local vascular microenvironment [9]. At the present time, components, which determine local vessel microenvironment, their interactions, and role in atherogenesis, are actively studying vessel wall biomechanics, arterial stiffness, and composition of extracellular matrix, local hemodynamic profile, etc.

Нypertension (HTN) and atherosclerosis have a complex synergistic effect, which modifies vascular microenvironment at all stages of cardiovascular disease continuum. However, studies focused on biomechanical factors in patients with HTN at various stages of atherogenesis are relatively rare; this dictates the necessity of the further clinical trials.

## 2. Aim

The aim is to evaluate carotid wall shear rate (WSR) in association with local and regional vascular stiffness in patients with hypertension (HTN) and atherosclerosis of peripheral arteries and to study the pattern of change of WSR in patients with HTN with increasing severity of peripheral artery atherosclerosis.

## 3. Materials and Methods

Study involved 133 patients with HTN, 65 men and 48 women, aged in average 57.9±10.8 years. Description of patients is given in Table 1.

Patients filled out the form of informed consent approved by local ethical committee. Clinical examination was performed; anamnesis data were collected. The following laboratory values were evaluated: total cholesterol (TC), triglycerides, high-density lipoprotein cholesterol (HDL-C), low-density lipoprotein cholesterol (LDL-C), creatinine with calculation of glomerular filtration rate (eGFR) by formula CKD-EPI, high-sensitivity C-reactive protein (hsCRP), and glycated hemoglobin (HbA1c).

Duplex scanning of carotid arteries and lower limb arteries with evaluation of intima-media thickness (IMT) of common carotid arteries (CCA) and common femoral arteries (CFA) was performed. Hemodynamics in examined arteries, the presence of plaque, and local stenosis of the vessel were evaluated [13]. Carotid IMT (CIMT) was calculated using automated (Auto IMT™, Samsung Medison EKO7, Korea) method. Mean CIMT (CIMT_m_) was defined as the arithmetic mean IMT of the left and right CCA IMT [14, 15]. The percentage of stenosis of the carotid arteries was assessed using ECST method. Maximal stenosis of carotid arteries was calculated [16]. Plaque score (PS) as summary height of all plaques in carotid arteries and total carotid plaque area (TCPA) as a summary area of all plaques in longitudinal position were calculated [17, 18].

Regional aortic stiffness was measured with Neurosoft Poly-Spectrum-PWV device by applanation tonometry method. Carotid-femoral (cfPWV) and carotid-radial (crPWV) pulse wave velocity (PWV) were measured. Investigation was performed in accordance with recommendations outlined in European expert consensus document on arterial stiffness and Russian expert consensus document on the evaluation of arterial stiffness in clinical practice [19, 20]. Local carotid stiffness was evaluated by carotid ultrasound. Systolic diameter (Ds) and diastolic diameter (Dd) of right CCA 1 cm proximal to the bifurcation of CCA were measured in M-mode. Final value of Ds and Dd was defined as mean value during a three cardiac cycle. The following indicators of local vascular stiffness were measured: distensibility coefficient (DC), distensibility (D), Peterson's elastic modulus (Ep), Young's elastic modulus (Ey), and stiffness index *β* (SI *β*). Abovementioned indicators were calculated by the following formulas [21–23]:

(1) DC=2ΔD×D+ΔD2ΔP×D2,D=ΔdΔP×ddEp=ΔP×DdΔD,Ey=ΔP×DdΔD×h,SI  β=ln⁡Ps×DdPd×ΔD,

where Δd is systolic-diastolic difference of CCA diameter, ΔP is difference in systolic and diastolic blood pressure, Dd is diastolic diameter of CCA, Ds is systolic diameter of CCA, Pd is diastolic blood pressure, and h id IMT.

During duplex ultrasound of carotid arteries peak blood flow velocity and diastolic vessel diameter were measured. In accordance with Hagen–Poiseuille law, WSS is determined by the formula [24]:

(2) $$\begin{array}{c} \tau =\frac{4\eta q}{\pi r3} \end{array}$$

where *τ* is WSS, *η* is blood viscosity, q is blood volume flow, and r is vessel radius.

According to formula (2), WSR can be determined by the formula:

(3) $$\begin{array}{c} \gamma =\frac{4\upsilon }{D} \end{array}$$

where *γ* is WSR, *υ* is peak blood flow velocity, and D is vessel diameter.

Measurements were made in the right CCA 1 cm proximal to the bifurcation of CCA in a place free from atherosclerotic plaques. Thus, the measurement of local carotid stiffness and WSR was carried out on the same part of the CCA. All studies were performed by one certified specialist.

Investigated cohort of patients was divided into groups in accordance with modified G. Belcaro et al. ultrasound morphologic classification of atherosclerotic vessel wall damage, which is presented in Table 2 [25, 26].

Each of four vessels (CCA bifurcation on both sides, CFA bifurcation on both sides) in each patient was assessed and received score according to the aforementioned classification.

Statistical analysis was performed using software IBM SPSS Statistics v. 22. Quantitative variables were described with the following statistics: mediana (Me) and 25th and 75th percentiles (LQ, UQ) in case of nonnormal distributed variables. For indicators with normal distribution, mean value (M) and standard deviation (SD) were used. Mutual impact of values was determined using Pearson's (in case of normal distributed variables) and Spearman's (for nonnormal distributed variables) correlation analysis. Integral equation describing dependence of carotid WSR on vascular stiffness parameters was formed using linear and multiple regression procedure. Pearson's chi-squared test and Kraskell–Wallis rank-based variance analysis were calculated with subsequent a posteriori calculation of the Mann–Whitney test. When comparing indicators obeying the normal distribution law, between more than two groups, the variance analysis was held, followed by the pairwise comparison, using the Bonferroni amendment. Differences were considered as statistically significant if error level p < 0.05.

## 4. Results

For features of various groups of patients, see Table 3.

In accordance with Table 3, the fourth group of patients had significantly smaller LDL cholesterol values in comparison with the first group of patients (p=0.002). In addition, the third and fourth groups of patients had significantly greater values of glycated hemoglobin in comparison with the first group of patients (p=0.029). The fourth group of patients was characterized by more statistically significant Ep values in comparison with the second (p=0.002) and the third (p=0.031) ones. CfPWV was less statistically significant in the first group of patients in comparison with the second (p=0.012) and the fourth groups (p=0.028) of patients. Ey values were significantly higher in the fourth group than in the second one (p=0.003). CCA distensibility in the fourth group of patients was significantly lower than in the first (p=0.015) and the second groups (p=0.003).

**Figure 1:**
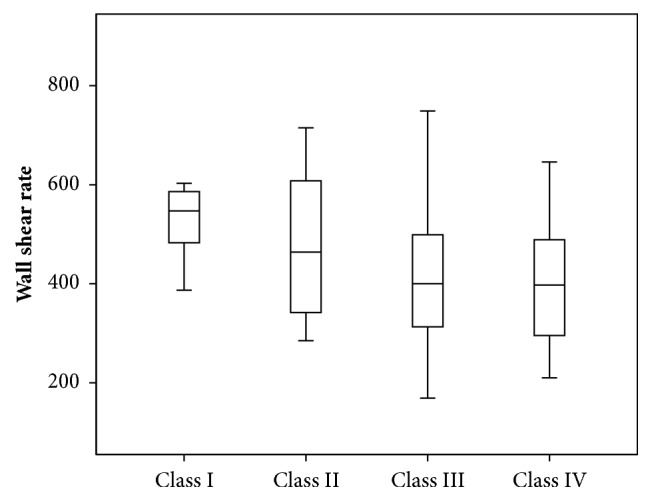
Wall shear rate in patients in different groups of patients.

During assessing of WSR in various groups of patients, the following results were obtained (see Figure 1).

The third and fourth groups of patients had WSR 416±128 s^−1^ and 405±117 s^−1^, respectively, which was significantly lower than the WSR in the first group of patients 546±112 s^−1^ (p_13_=0.002; p_14_=0.004). The values of WSR in the second group of patients were 478±151 s^−1^, which were not significantly different from the values in other groups of patients.

Decreased carotid WSR was associated with increased Ep (r=-0.229; p=0.021), decreased DC (r=0.294; p=0.031), and D (r=-0.332; p=0.0001). Decreased WSR was also associated with increased aortic stiffness (r=-0.368; p=0.001) and regional muscular vessels stiffness, which was assessed by crPWV (r =-0.251; p=0.032). For parameters of linear regression equations predicting carotid WSR changes depending on various parameters of local and regional vascular stiffness, see in Table 4.

As Table 4 shows, according to the results of linear regression, it was established that CCA distensibility, as an indicator of local carotid stiffness, and cfPWV, as indicator of regional vascular stiffness, are maximally contributed to the variability of WSR. CCA distensibility value (x) can be used to give WSR (y) with equation of model received with regression analysis: y = 0.332 *∗* x + 357.19. The obtained model is significant (p=0.0001). However, only 11.0% WSR variability can be attributed to variability of CCA distensibility. Decreasing of CCA distensibility by unit is an indication of WSR decreasing by 0.332 s^−1^. The variability of cfPWV can account for 13.6% variability of carotid WSR. Equation of model is as follows: y = – 18.546 *∗* x + 645.45. Angular coefficient is – 18.546 (CI [-29.627;-7.465]). That is, increasing of cfPWV by unit is an indication of shear rate decreasing by 18.546 s^−1^.

**Figure 2:**
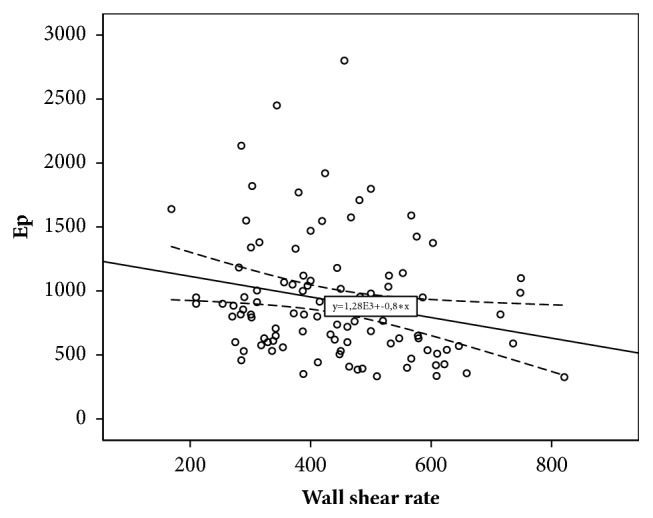
The relationship between wall shear rate and Peterson's elastic modulus.

**Figure 3:**
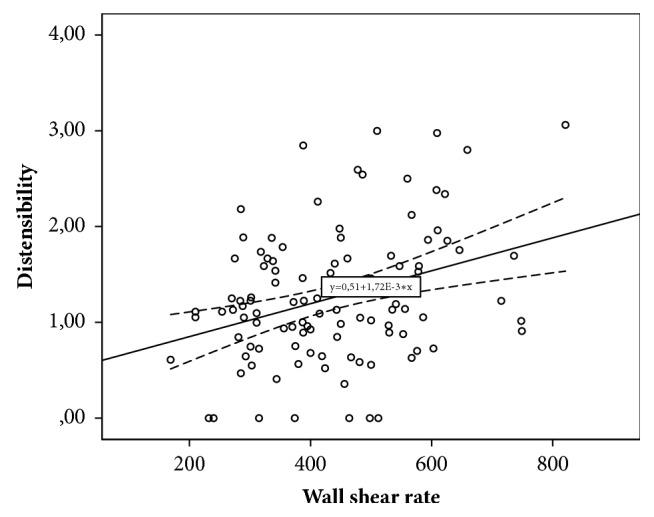
The relationship between wall shear rate and carotid distensibility.

**Figure 4:**
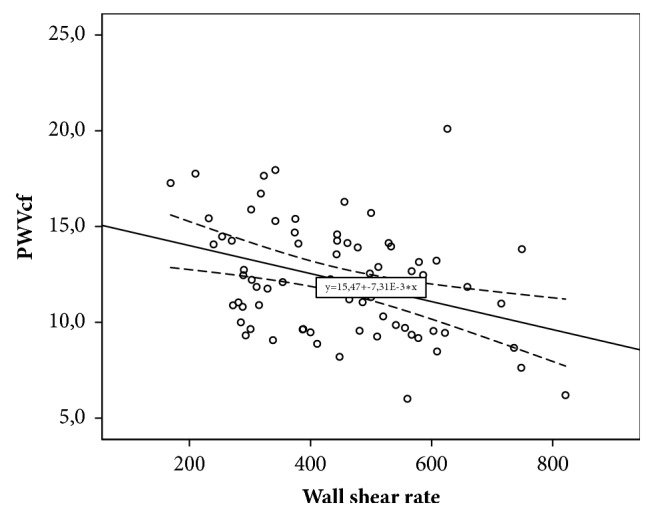
The relationship between wall shear rate and carotid-femoral pulse wave velocity.

**Figure 5:**
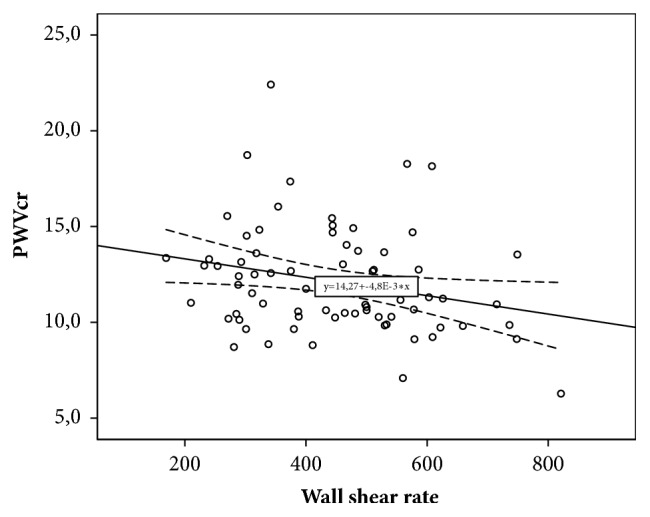
The relationship between wall shear rate and carotid-radial pulse wave velocity.

For scatter diagrams with marked graphical components of regression analysis, see in Figures 2–5.

Besides this, values of WSR were related to ultrasound parameters that characterize severity of carotid atherosclerosis. The low values of WSR were associated with maximal stenosis of carotid arteries (r=-0.275; p=0.01), stenosis of carotid arteries (r=-0.247; p=0.01), PS (r=-0.242; p=0.015), and TCPA (r=-0.220; p=0.043). Decreased WSR was also associated with patients in older age group (r=-0.360; p=0.001) and with type 2 DM (r=-0.274; p=0.006).

Further multiple regression analysis was performed to exclude the influence of confounding factors. The analysis was performed with adjustments to factors such as sex, age, type 2 DM, and therapy (statins and hypotensive drugs). The results are shown in Table 5.

Thus, based on the results of multiple regression analysis, the relationships between WSR and such local carotid stiffness parameters as Ep and D remained statistically significant. According to results of a multifactor analysis interrelations between regional vascular stiffness and shear rate have lost statistical significance.

## 5. Discussion

It has been evidenced that biomechanical forces and factors are essential at all stages of atherogenesis. It is necessary to highlight the complexity of such value as WSR. Firstly, WSR is an adequate surrogate marker of the WSS that allows assessing it indirectly without direct invasive evaluation of blood viscosity. Secondly, WSR determines the time at which the blood components are retained over the site of the vascular wall. Thus, WSR determines local concentration of atherogenic substances and velocity of chemical reactions in vessel wall. With decreasing of WSR, time that atherogenic blood components exposed to vessel wall is increasing, which is potentially atherogenic factor [27]. In 2016, the results of prospective study conducted by C. Carallo et al. were published. This study was the first established predictive value of carotid WSS related to plaque occurence, which confirms that study of WSR is clinically significant [28]. Apart from that, number of studies has established correlation between decreased WSR measured in various vascular beds in patients with coronary atherosclerosis and peripheral arterial atherosclerosis [29–32].

In our study, it was established that in patients with HTN carotid WSR progressively decreased with increasing of atherosclerotic lesions in peripheral arteries severity. HTN triggers short-term and long-term adaptation mechanisms known to increase arterial wall thickness and to change vessel diameter. HTN is more often accompanied with increasing of internal and external diameters in elastic and muscular-elastic arteries [33]. One consequence of these processes is decreasing of WSS and WSR in accordance with Hagen–Poiseuille law. With progression of atherosclerosis and plaque formation, cross-sectional area of the lumen is increasing, and that again contributes to further decreasing of WSR. However, as has been shown by Stiel et al., a compensatory enlargement of the vessel diameter is possible only in the early stages of the atherosclerotic process, until the development of significant lumen stenosis [34]. With an increase in the degree of stenosis of more than 75% area and 50% diameter a further increase in the size of the vessel is not observed and its compensatory enlargement is transformed into obstruction. It can stabilize WSS and WSR or to their increasing. The results we received partly illustrate pattern of WSR changes in patients with HTN and atherosclerosis of peripheral arteries.

In literature, there are a small number of dates consistent with results we received. In the study conducted by R. Duivenvoorden et al., the low WSS, which was assessed with phase contrast magnetic resonance angiography, associates with decreasing of carotid artery compliance [35]. In study conducted by M. W. Rajzer et al., decreased WSS in ascend aorta was associated with increasing of cfPWV, which corresponds to aortic rigidity [36]. M. Schäfer et al. studied interrelation between WSS and parameters of local vascular stiffness in patients with pulmonary hypertension. It has been established that decreased WSS in pulmonary artery was associated with increased pulmonary artery stiffness assessing by such parameters as distensibility and Peterson's elastic modulus [37]. In our study involved with mixed population of patients, decreased carotid WSR was directly correlated with increased local vascular stiffness as well as regional vascular stiffness at aortic level. In addition, contribution of local and regional vascular stiffness values to the distensibility of carotid WSR was assessed. Earlier, WSS and WSR were considered only as local phenomenon describing only limited part of vascular bed. Nevertheless, correlation of WSR measured in carotid artery to the aortic stiffness calls into question this statement.

On the other hand, the absence of statistically significant interrelations between the indices of regional vascular stiffness and the WSR according to multivariate analysis requires further study. Age, diabetes, and hypertension are known to be the main determinants of aortic stiffness. This probably explains the results of the multiple regression analysis.

## 6. Conclusion

In patients with HTN and atherosclerotic lesions of peripheral arteries, it is registered that the carotid WSR decreased with increasing severity of atherosclerosis. Decreased carotid WSR is associated with increased local carotid stiffness, regional vascular stiffness of muscular, and elastic vessels.

## Figures and Tables

**Table 1:** Clinical and laboratory characteristics of patients.

Characteristics	Patients (n=113)
Age (years, M±SD)	57.9±10.8
Smoking (n, %)	37 (33.7%)
BMI (kg/m^2^, Me (LQ; UQ))	26.0 (29.0; 31.0)
Coronary artery disease (n, %)	75 (66.4%)
Myocardial infarction (n, %)	37 (33.7%)
Coronary artery revascularization (n, %)	41 (36.3%)
Stroke (n, %)	6 (5.31%)
Intermittent claudication, (n, %)	17 (15.0%)
Type 2 diabetes mellitus (n, %)	41 (36.3%)

Laboratory findings	

TC (mmol/l, M±SD)	4.83±1.15
LDL-C (mmol/l, M±SD)	2.80±1.09
HDL-C (mmol/l *π*, Me [LQ; UQ])	1.20 [1.04; 1.57]
Triglycerides (mmol/l, Me [LQ; UQ])	1.55 [1.07; 2.01]
eGFR (ml/min/1.73 m^2^, Me [LQ; UQ])	60.0 [53.5; 70.2]
hsCRP (mg/l, Me [LQ; UQ])	1.71 [0.84; 4.37]
HbA1c (%, Me [LQ; UQ])	5.18 [4.50; 5.70]

Medication	

Antiplatelets (n, %)	91 (80.5%)
Statins (n, %)	70 (61.9%)
ACE inhibitors (n, %)	80 (70.8%)
Beta-blockers (n, %)	65 (57.5%)
Diuretics (n, %)	18 (15.9%)
Calcium channel blockers (n, %)	9 (7.96%)
Oral antidiabetic medications (n, %)	25 (22.1%)

BMI: body mass index; TC: total cholesterol; HDL-C: high-density lipoprotein cholesterol; LDL-C: low-density lipoprotein cholesterol; eGFR: estimated glomerular filtration rate; hsCRP: high-sensitivity C-reactive protein; ACE: angiotensin-converting-enzyme.

**Table 2:** Ultrasound morphologic classification of atherosclerotic vessel wall.

Class	Ultrasound Morphology	Score
I	Normal: 3 ultrasonic layers clearly separated; CIMT ≤ 0.9 mm	2

II	Intima-media granulation: granular echogenicity of deep, normally anechoic intimal-medial layer and/or increased intima-media thickness (≥0.9 mm)	4

III	Plaque without hemodynamic disturbance: focal structures encroaching into the arterial lumen of at least 0.5 mm or 50% of the surrounding CIMT value, or CIMT >1.5 mm	6

IV	Stenotic plaque: as in class III, but with hemodynamic stenosis on duplex scanning, indicating stenosis >50%	8

CIMT: carotid intima-media thickness.

**Table 3:** Clinical, laboratory, and instrumental characteristics of patients in four groups.

Characteristics	Group I (n=15)	Group II (n=11)	Group III (n=60)	group IV (n=26)	p
Age (years, M±SD)	43.9±11.8	54.4±9.56	60.2±9.04	62.1±8.07	0.0001
CAD (n)	4	6	41	24	0.0001
DM type 2 (n)	5	1	19	16	0.023
LDL-H (mmol/l, M±SD)	2.73±0.83	3.25±1.24	2.68±1.14	2.78±1.09	0.454
HDL-H (mmol/l, Me [LQ; UQ])	1.63 (1.34; 1.72)	1.22 (1.11; 1.45)	1.21 (1.07; 1.56)	1.06 (0.97; 1.17)	0.002
TG (mmol/l, Me [LQ; UQ])	0.98 (0.85; 1.36)	1.74 (1.46; 1.88)	1.54 (1.07; 2.23)	1.69 (1.25; 2.05)	0.103
eGFR (ml/min/1.73 m^2^, Me [LQ; UQ])	65.7 (56.4; 74.9)	67.2 (55.2; 68.1)	59.1 (52.7; 70.2)	60.7 (55.8; 68.6)	0.571
hsCRP (mg/l, Me [LQ; UQ])	1.35 (0.49; 3.07)	1.57 (0.92; 3.92)	2.33 (0.93; 5.22)	1.65 (0.81; 4.58)	0.655
HbA1c (%, Me [LQ; UQ])	4.50 (4.00; 5.10)	4.80 (4.55; 5.50)	5.20 (4.70; 5.60)	5.55 (4.57; 7.17)	0.026
CIMT_m_, (mm, M±SD)	0.70±0.08	0.92±0.15	0.86±0.16	0.97±0.10	0.0001
PS, (mm, Me [LQ; UQ])	0.00 (0.00; 0.00)	0.00 (0.00; 0.00)	3.00 (2.00; 4.68)	4.30 (3.71; 6.10)	<0.0001
TCPA, (mm^2^, Me [LQ; UQ])	0.00 (0.00; 0.00)	0.00 (0.00; 0.00)	0.31 (0.16; 0.42)	0.59 (0.44; 0.64)	<0.0001
Maximum stenosis of CA (%, Me [LQ; UQ])	0.00 (0.00; 0.00)	0.00 (0.00; 0.00)	35.0 (26.0; 40.0)	50.0 (39.7; 55.7)	<0.0001
DC (10^−3^/mmHg, M±SD)	3.02±1.33	3.58±0.78	2.51±1.02	1.99±0.98	0.235
D (mmHg^−1^*∗*10^3^, M±SD)	1.66±0.75	1.65±0.69	1.21±0.60	1.01±0.50	0.004
Ep (mmHg, M±SD)	743±389	592±193	882±380	1193±502	0.002
Ey (mmHg/mm, M±SD)	1225±472	726±286	1128±502	1515±610	0.012
SI *β* (M±SD)	8.04±3.46	6.84±1.52	8.64±3.77	10.7±4.20	0.080
cfPWV (m/s, M±SD )	9.35±2.31	11.8±2.55	12.7±2.89	13.0±2.06	0.008
crPWV (m/s, M±SD )	10.7±2.56	12.9±4.49	12.4±2.50	11.9±2.06	0.591

CAD: coronary artery disease; TC: total cholesterol; HDL-C: high-density lipoprotein cholesterol; LDL-C: low-density lipoprotein cholesterol; eGFR: estimated glomerular filtration rate; hsCRP: high-sensitivity C-reactive protein; mean carotid intima-media thickness; DC: distensibility coefficient; D: distensibility; Ep: Peterson's elastic modulus; Ey: Young's elastic modulus; SI *β*: stiffness index *β*; cfPWV: carotid-femoral pulse wave velocity; crPWV: carotid-radial pulse wave velocity.

**Table 4:** Parameters of linear regression equations.

Characteristics	R	R^2^	B	95% CI for B	р
Ep	0.229	0.052	-0.065	(-0.120) - (-0.100)	0.021
Index-*β*	0.147	0.022	-4.288	(-10.157) -1,581	0.150
D	0.332	0.110	0.332	0.287 – 0.991	0.0001
crPWV	0.251	0.063	-13.142	(-25.122) - (-1.163)	0.032
cfPWV	0.368	0.136	-18.546	(-29.627) - (-7.465)	0.001

Ep: Peterson's elastic modulus; SI *β*: stiffness index *β*; D: distensibility; cfPWV: carotid-femoral pulse wave velocity; crPWV: carotid-radial pulse wave velocity.

**Table 5:** Parameters of multiple regression analysis.

Characteristics	R	R^2^	B	95% CI for B	р
Ep	0.321	0.103	-0.288	(-0.138) – (-0.018)	0.012
Index-*β*	0.470	0.221	-0.135	(-9.82) – 2.38	0.227
D	0.511	0.261	0.257	0.239-0.887	0.005
crPWV	0.604	0.365	-0.129	(-18.80) – (6.05)	0.306
cfPWV	0.539	0.352	-0.55	(-18.36) – (12.89)	0.726

Ep: Peterson's elastic modulus; SI *β*: stiffness index *β*; D: distensibility; cfPWV: carotid-femoral pulse wave velocity; crPWV: carotid-radial pulse wave velocity.
